# The epidemiology of tuberculosis in Phuentsholing General Hospital: a six-year retrospective study

**DOI:** 10.1186/1756-0500-5-311

**Published:** 2012-06-20

**Authors:** Kinley Wangdi, Manish Raj Gurung

**Affiliations:** 1 Phuentsholing General Hospital, G.P.O. Phuentsholing, Chhukha, Bhutan

## Abstract

**Background:**

This study was carried out to describe the epidemiology and treatment outcomes of TB infection in Bhutan at Phuentsholing General Hospital (PGH). Retrospective analysis of TB data was carried out using data from the TB registry of PGH from 2004–2009. Comparisons were made between TB, clinical presentation, diagnosis, and outcomes amongst male and female.

**Findings:**

A total of 735 patients were analyzed, 12.4% (91) of whom were children (≤14 years). The highest cases was reported in 2009 (148), lowest in 2004 (93). Males and females were equally infected with TB. The median age was 25 years, (range 11 months - 98 years; IQR = 20-35). Extra-pulmonary Tuberculosis (EPT) 62.6% (57) was the commonest form of TB in children. Pleural effusion was more common in males 62.8% (27) (*p* = 0.013). Smear positive pulmonary tuberculosis (SPPT) 54.3% (207) (*p* = 0.02) and treatment defaulted 84.2% (16) (*p* = 0.004) was higher in males. However, transfer-in cases 57.0% (90) (*p* = 0.036) and treatment outcome-failure 92.3% (12) (*p* = 0.002) were more in females than males. The cure rate for SPPT was 69.0% (293) and unknown treatment outcome for all forms of TB was 11.2% (82).

**Conclusion:**

TB infection has increased over the study period; SPPT increased more than other two forms of TB. The majority of the TB patients were in the age group of 15–34 years. Males and females were equally infected with TB and children made up 12.4% of TB patients. The cure rate amongst SPPT was 69%, which is much lower than the national target of 85% set by National Tuberculosis Control Programme (NTCP). Further studies need to be undertaken to identify the risk factor for TB in the economically productive age group. There is a need for improvement in the services, recording and reporting so as to meet the target of cure rate of 85% in SPPT patients.

## Background

Tuberculosis (TB) is a public health problem caused by *Mycobacterium tuberculosis*. World Health Organization (WHO) estimates that there are almost 13.7 million people living with TB and that the disease kills more young people and adults than any other infectious disease in the world [1]. A total of about 1.77 million people died of TB in 2007 including 456,000 patients infected with the Human Immunodeficiency Virus (HIV). TB is the seventh most common disease in the world and is the leading cause of death from curable diseases [2]. WHO declared TB a global health emergency in 1993 following dramatic changes in the magnitude of the problem. However, the number of new cases continues to increase and without a coordinated control effort, it is estimated TB will infect more than one billion people by 2020, with deaths reaching 36 million [3]. Various interrelated factors have been attributed to the resurgence of TB in recent years, including the HIV epidemic, ineffective control-programmes, population growth, and increased mobility of people [1,4-7].

In the South-East Asia Region (SEAR) of WHO, the annual incidence of TB is 3.17 million cases, with an estimated 4.88 million prevalent cases [8]. This amounts to one-third of the global burden of TB. Five of the 11 member countries in the region are among the 22 high-burden countries, with India accounting for over 20% of the world’s cases. Most cases occur in the age group of 15–54 years, with males being disproportionately affected. The male/female ratio among newly detected cases was 2:1. The deaths as a result of TB have declined after introduction of Directly Observed Treatment Short course chemotherapy (DOTS).

This study was carried out to describe the epidemiology and treatment outcomes of TB infection in Phuentsholing General Hospital (PGH). Tuberculosis disease, clinical presentation, diagnosis and outcomes amongst males and females were compared. The study was carried over six-month duration, from January to July 2011.

## Methods

### Setting

Phuentsholing General Hospital (PGH) is located in the Phuentsholing Municipality, under Chukha Dzongkhag. Phuentsholing Municipality is the second largest city, next to the capital city, Thimphu, in Bhutan. It is located 171 km south of the capital city, and it is the commercial hub of Bhutan. PGH is a fifty-bed hospital, with specialist services, including General Surgery and Obstetrics and Gynaecology, besides the general patient care. The hospital provides health care services to the people residing in Phuentsholing Municipality and other sub-districts of Phuentsholing and Sampheling; however, the population of two other sub-districts, Lochina and Dungna, also avail the diagnostic services at PGH.

### Tuberculosis control in Bhutan

The National Tuberculosis Control Programme (NTCP) was initiated in 1976 [9,10]. NTCP is one of the programmes under the Department of Public Health. The NTCP has incorporated global targets and Millennium Development Goals (MDG) and developed the following objectives: (1) detect at least 70% of the estimated new smear-positive cases, (2) cure at least 85% of the newly diagnosed cases, (3) have halted and begun to reverse incidence of TB by 2015, and (4) halve the prevalence and death rate of TB between 1990 and 2015. Bhutan adopted the DOTS strategy in 1997. TB control and treatment was integrated into the general health care delivery system [9-11]. In line with the DOTS strategy, diagnosis is primarily based on sputum smear and chest x-ray. The resources for accurate diagnosis of TB are limited, and culture and drug susceptibility testing for *M. tuberculosis* have not been performed routinely.

The treatment regimen in Bhutan underwent many changes from 2004 to 2010 [9,10]. Before 2006, new SPPT and smear negative pulmonary tuberculosis (SNPT) seriously ill were treated with four drugs (streptomycin, isoniazid, rifampicin, and pyrazinamide) for two months of intensive therapy, and two drugs (isoniazid and ethambutol) in continuation therapy for another six months. SPPT relapse and failure cases were treated with five drugs (streptomycin, isoniazid, rifampicin, ethambutol and pyrazinamide) for two months of intensive therapy and four drugs (isoniazid, rifampicin, ethambutol and pyrazinamide) for one month, followed by three drugs (isoniazid, rifampicin, and ethambutol) for five months of continuation therapy. EPT was treated with three drugs (isoniazid, rifampicin, and pyrazinamide) in intensive therapy and two drugs (isoniazid and ethambutol) for six months in continuation therapy. However, since 2007, all new TB cases irrespective of their forms were treated with four drugs (isoniazid, rifampicin, ethambutol, and pyrazinamide) for two months of intensive therapy, and two drugs (isoniazid and ethambutol) in continuation therapy for another six months. In 2009, PGH contributed 9.9% of the total TB cases (1,154) in Bhutan [12].

### Study design and data collection

This study was carried out retrospectively using the data from the TB registry of PGH from 2004 to 2009. All the patients taking the anti-tuberculosis treatment (ATT) were recorded in the TB register and subsequently reported to the NTCP. Patients with symptoms such as fever and cough lasting more than three weeks, chest pain, haemoptysis, loss of weight, and loss of appetite, those suggestive of TB, submitted three sputum samples: one spot and two on the subsequent days. Patients with at least two positive smears or one positive smear and radiologic abnormalities consistent with pulmonary tuberculosis, or one positive smear and sputum culture positive were considered to have SPPT. Those patients with three negative smears were treated with a course of antibiotic and evaluated subsequently. These latter patients were considered to have SNPT if they presented with a chest X-ray suggestive of pulmonary TB and did not response to antibiotics. The diagnosis of EPT was made based on the radiological, histological, or clinical evidence consistent with active TB in an extra-pulmonary site.

Patients diagnosed with SPPT were admitted to the hospital for duration of two months for intensive therapy. All the patients were included in DOTS. Patients in other categories of TB- SNPT and EPT were admitted if the patients were ill or if they inhabited remote villages. All patients were offered anti-tuberculosis drugs free of charges because all the health care in Bhutan is provided free of charge. The patients underwent two months of intensive therapy followed by six months of a continuation phase. All TB patients submitted sputum samples for smear examination for acid fast bacilli (AFB) conversion at the end of second, fifth, and eight months, respectively.

Once patients were diagnosed as having TB, they were sent to the TB In-charge for recording and reporting. The information recorded in the TB register included: name, age, gender, address, type of infection (new, relapse, default, treatment failure, transferred in), type of TB (SPPT, SNPT, and EPT), treatment regimen, sputum conversion (2, 5, and 8 months), and treatment outcome (cured, completed treatment, defaulter, failure of treatment, died, or transferred out).

### Study population

The study population for the present study was all the TB patients registered in PGH hospital from 2004 to 2009.

### Statistical analysis

Data analysis was carried out using SPSS (version 10 for Microsoft Windows). Descriptive statistics (frequencies and means) were used to describe the demographic characteristics and treatment outcomes. Chi-square test and Fisher Exact test were used to find the association of different variables between male and female patients. The *p*-value of less than 0.05 was considered as significant.

### Ethical issues

The ethical approval was obtained from the Research Ethical Committee of Health (RECH), Ministry of Health, Bhutan. To protect the privacy of the patients, the names of the patients were not included in case record form. Hospital numbers were not directly recorded, and it was delinked from the registration by using alternative number. The alternative number was used when some information was not entered correctly.

## Findings

The total number of patients who received treatment for all forms of TB during the study period was 745. Due to the missing or incomplete information in 10 records, the cases that were included for the study were 735. There was gradual increase in the number of cases over the study period with the highest cases reported in 2009 (148 cases) and the lowest number was recorded in 2004 (93 cases). SPPT increased from 36.6% (34) in 2004 to 60.8% (90) in 2009. However, the other two forms of TB; SNPT and EPT, decreased over the study period (Table 1).

**Table 1:** Different forms of tuberculosis (TB), cases definitions, TB among gender, TB among the children and treatment outcome

	**2004**	**2005**	**2006**	**2007**	**2008**	**2009**
Tuberculosis in males (%)	44 (47.1)	49 (46.7)	76 (57.1)	63 (47.7)	67 (54.0)	70 (47.3)
TB cases by different age groups (years) n (%)						
0-14	20 (21.5)	19 (18.1)	16 (12.0)	12 (9.1)	10 (8.1)	14 (9.5)
15-24	18 (19.4)	31 (29.5)	45 (33.8)	46 (34.8)	53 (42.7)	54 (36.5)
25-34	21 (22.6)	25 (23.8)	43 (32.3)	36 (27.3)	35 (28.2)	50 (33.8)
35-44	11 (11.8)	11 (10.5)	9 (6.8)	15 (11.4)	13 (10.5)	11 (7.4)
45-54	9 (9.7)	9 (8.6)	8 (6.0)	13 (9.8)	32 (2.4)	6 (4.1)
≥55	14 (15.1)	10 (9.5)	12 (9.0)	10 (7.6)	10 (8.1)	13 (8.8)
All forms of TB (n)	93	105	133	132	124	148
SPPT (%)	34 (36.6)	45 (42.9)	58 (43.6)	73 (55.3)	81 (65.3)	90 (60.8)
SNPT (%)	24 (25.8)	18 (17.1)	35 (26.3)	25 (18.9)	7 (5.7)	20 (13.5)
EPT (%)	35 (37.6)	42 (40.0)	40 (30.1)	34 (25.8)	36 (29.0)	38 (25.7)
Case definitions						
New cases (%)	81 (87.4)	68 (64.8)	96 (72.2)	84 (63.6)	80 (64.5)	103 (69.6)
Relapse (%)	2 (2.2)	4 (3.8)	2 (1.5)	13 (9.9)	13 (10.5)	7 (4.7)
Transfer in (%)	9 (9.9)	33 (31.4)	30 (22.6)	30 (22.7)	24 (19.4)	32 (21.6)
Treatment after default (%)	1 (1.1)	0 (0)	2 (1.5)	3 (2.3)	1 (0.8)	6 (4.1)
Failure (%)	0 (0)	0 (0)	0 (0)	1 (0.8)	0 (0)	0 (0)
Others (%)	0 (0)	0 (0)	1 (0.8)	0 (0)	0 (0)	0 (0)
Unknown (%)	0 (0)	0 (0)	2 (1.5)	1 (0.8)	6 (4.8)	0 (0)

SPPT- sputum positive pulmonary tuberculosis; SNPT- sputum negative pulmonary tuberculosis; EPT-extra-pulmonary tuberculosis.

Regarding the types of infection, new cases gradually reduced over the study period: 87.4% (81) in 2004, reducing to 69.6% (103) in 2009. Relapse case increased gradually over the study period, with highest number of cases reported in 2008 (10.9%). Transfer-in cases increased from 9.9% (9) in 2004 to 21.9% (32) in 2009. The proportion of males to females remained the same throughout the study period, with equal numbers of males and females succumbing to TB. TB in children decreased from 21.5% (20) in 2004 to 9.5% (14) in 2009. Extra-pulmonary Tuberculosis (EPT) 62.6% (57) was the commonest form of TB in children. The median age was 25 years, and the age range was 11 months to 98 years (IQR = 20-35). The highest number of TB cases was reported among the 15–34 year-old age group 62.2% (457).

### TB distribution between males and females

TB between male and female was compared to investigate any differences in types of the disease, forms of TB, and treatment outcomes (Table 2). There were no differences between males and females for new cases and relapses. However, the number of transfer-in cases was higher among females than males, with 57.0% (90) to 43.0% (68) (*p* = 0.036). SPPT was more common among males than females with 54.3% (207) to 45.7% (174) (*p* = 0.02). There was no difference of SNPT in either gender (*p* = 0.3). When different forms of EPT were compared; only pleural effusion was more common in males than females 62.8% (27) to 37.2% (16) (*p* = 0.013). When comparing the treatment outcome among male and female,“Failures” and “defaulters” were observed to be significantly different between males and females. There were more treatment failures in females than males 92.3% (12) to 7.7% (1) (*p* = 0.002); however, there were more male treatment defaulters than females 84.2% (16) to 15.8% (3) (*p* = 0.004).

**Table 2:** TB distribution between female and male by case definitions forms of TB and treatment outcome

	**Total**	**Female (%)**	**Male (%)**	**P value**
Case definitions				
New cases	512	246 (48.1)	266 (52.0)	0.2
Relapse	41	18 (43.9)	23 (56.1)	0.5
Transfer in cases	158	90 (57.0)	68 (43.0)	0.036
Treatment after default	13	5 (38.5)	8 (61.5)	0.58
Forms of TB				
SPPT	381	174 (45.7)	207 (54.3)	0.02
SNPT	129	70 (54.3)	59 (45.7)	0.3
EPT				
TB lymphadenitis	43	26 (60.5)	17 (39.5)	0.36
TB osteomyelitis	7	2 (28.6)	5 (71.4)	0.25
Pleural effusion	43	16 (37.2)	27 (62.8)	0.013
Treatment outcome				
Cured	263	127 (48.3)	136 (51.7)	0.54
Treatment completed	333	169 (50.8)	164 (49.3)	0.64
Death	15	9 (60.0)	6 (40.0)	0.43
Failure	13	12 (92.3)	1 (7.7)	0.002
Defaulted	19	3 (15.8)	16 (84.2)	0.004
Stopped	4	2 (50.0)	2 (50.0)	1.0
Transferred out	6	4 (66.7)	2 (33.3)	0.45
Outcome unknown	82	40 (48.8)	42 (51.2)	0.85

SPPT- sputum positive pulmonary tuberculosis; SNPT- sputum negative pulmonary tuberculosis; EPT-extra-pulmonary tuberculosis.

### Treatment outcome by types of infection

The treatment outcomes of different forms of TB were compared (Table 3). The treatment cure rate for the SPPT cases was 69.0% (263) during the study period. Treatment completed was achieved in 16.3% (62) of SPPT, 70.5% (91) of SNPT, and 80.0% (180) of EPT cases. The defaulter rate from all forms of infection during the study was 2.6% (19). However, defaulters were highest for the SNPT 3.1% (4). Mortality from all forms of TB was 2.0% (15); the highest death rate was observed among SNPT patients 3.1% (4). Treatment failure rate was highest for SPPT at 2.9% (11). Treatment “outcome unknown” for all types of TB during the study period was 11.2% (82), and highest rate was reported for SNPT 20.2% (26).

**Table 3:** Treatment outcome by types of infection

	**PTB**				**EPT**		**All forms of TB**	
	**SPPT**		**SNPT**				**Total**	
	**n**	**%**	**N**	**%**	**n**	**%**	**n**	**%**
Completed	62	16.3	91	70.5	180	80.0	333	45.3
Cured	263	69.0	0	0	0	0	263	35.8
Defaulted	11	2.9	4	3.1	4	1.8	19	2.6
Died	7	1.8	4	3.1	4	1.8	15	2.0
Failure	11	2.9	1	0.8	1	0.4	13	1.8
Stopped	0	0	2	1.6	2	0.9	4	0.5
Transferred out	5	1.3	1	0.8	0	0	6	0.8
Unknown	22	5.8	26	20.2	34	15.1	82	11.2
Total	381	100.0	129	100.0	225	100.0	735	100.0

SPPT- sputum positive pulmonary tuberculosis; SNPT- sputum negative pulmonary tuberculosis; EPT-extra-pulmonary tuberculosis.

## Discussion

There was a gradual increase of TB over the study period. There was a significant increase in SPPT, with decrease in the other forms of TB, SNPT and EPT, over the six-year period. A number of plausible risk factors for TB infection such as, HIV infection, smoking, alcohol use, and other life style associated diseases such as diabetes and hypertension, malignancies, and chronic lung diseases have been reported in other researches, but could not be covered in this study [13-15]. However, some of plausible reasons for increased TB cases in PGH could be rapid urbanization and crowding, with related poor sanitation and malnutrition. There is inadequate housing in Phuentsholing, with many people residing in the Indian town of Jaigon, West Bengal. Poor socioeconomic conditions have been identified as the main risk factors associated with the transmission of TB [16]. Other reasons, such as increased awareness of TB, could have lead to more people seeking medical care. Improved health care services with improved diagnostic facilities for TB may also contribute to the higher number of detected cases. Similar reasons have been discussed by Jetan et al. in Malaysia [17]. People living with HIV/AIDS (PLWHA) in Phuentsholing are second highest number of those infected in the country. HIV increases the susceptibility to TB by many times [18]. This could be one of the plausible reasons for increasing TB cases in Phuentsholing. However, HIV status of the TB patients under study was not available, despite all TB patients were offered voluntary counselling and testing (VCT) services at the hospital.

The majority of cases 62.2% (457) were in the 15–34 year-old age group. The younger people are increasingly changing their life style and are involved in unhealthy lifestyle and practices, such as eating junk and less nutritious food, increased use of alcohol, tobacco and drugs thus providing opportunities for latent TB to reactivate into active infection. However, the underlying risk factor for increased TB in age group 15-34 years was not covered in the present study and it is imperative that these risk factors be explored further in future so that programme can develop strategies and policies to reduce TB infection in this age group. Jeten et al. reported that the higher cases of TB among the productive age group were a result of employment, whereby infection was exposed at the work place [17]. This could be possible in Phuentsholing as well since there are huge employment opportunities in Pasakha Industrial Estate and Phuentsholing town. Children made up to 12.4% of TB cases during the study period. The finding that children presented commonly with EPT 62.6% is consistent with other studies, and it has been associated with the difficulty in collection of sputum in children [19].

### TB distribution between males and females

There were equal numbers of males and females succumbing to TB. Similar findings have been reported in the literature, suggesting that the access to and use of healthcare services is similar in men and women [20]. While other research has reported higher male compared with female TB infection due to differential health seeking behaviour, stigma associated to TB infection, and barriers to health care [21-23].

Higher rates of SPPT among males compared with females were noted in the present study. Some of the plausible reasons could be as a result of infection acquired outside in a crowded, poorly ventilated work setting. The male population usually work outside such as farms and factories, while females work at the home. Males are also more likely to report risk factors such as frequent use of alcohol and tobacco, important deterrents for latent TB infection progression to active TB. However, these risk factors were not covered in the present study. More transfer-in cases were observed among females. This could be as a result of the people migrating to Phuentsholing for businesses and job opportunities as explained earlier because Phuentsholing is the commercial hub of Bhutan. Males were more likely to default from treatment, and similar findings had been found in other studies [20]. Higher cases of pleural effusion were found among men in this study; however, other studies showed that pleural effusion was more common in females, contrary to the findings of this study [24]. Other studies reported higher defaulter and failure rates among males; however, in this studies treatment failure were more common in females whereas, the defaulters observed to be higher among males [20].

### Treatment outcomes

The treatment outcome - cured for new SPPT cases during the study was 69.0%, which is lower than the national target of 80% and the national cure rate of 89% in 2007 [9,10,25]. Some of the reasons for not being able to achieve the national target could be poor follow-up as a result of gaps in human resources as well as due to the migrant nature of the population/floating population availing the services from PGH. High treatment failure in SPPT needs to be further explored because this can lead to development of multi-drug resistant (MDR) TB. There was a significant percentage 11.2%, with unknown treatment outcome, which indicated poor follow-up and default tracing.

### Limitations

These findings were subject to at least five limitations. First, sociological data were not available, and the information that was available, recovered from registers, may be imprecise in some cases. Second, the present study was unable to access the available data on HIV status. Third, there is no facility for drug sensitivity test (DST). Fourth, the treatment regimens changed during the period of study, and this may contribute to the results found. Fifth, some of the findings of this study may not necessarily reflect national trends because the study was based on the patients diagnosed and treated in PGH.

## Conclusions

In conclusion, there was a gradual increase in TB infection over the study period. The increase was more in SPPT as compared to other two forms of TB-SNPT and EP. New cases represented the majority of patients over the study period. The majority of the TB patients were in the economically productive age group of 15–34 years. There is a need to identify the underlying cause for this finding. This could enable the programme to develop strategies and policies to reduce TB infection in the age group of 15–34 years. Equal numbers of males and females succumbed to TB. SPPT was more common among males than females while there was no difference of SNPT in either gender. Pleural effusion was more common in males than females when EPT was compared. Treatment failures were more in females than males, while more males defaulted from treatment. Children made up 12.4% of TB patients. The cured rate amongst SPPT was 69%, much lower that the national target of 85% set by NTCP. The treatment outcome unknown was 11.2% indicating poor follow up and monitoring. There is an urgent need for improvement in the recording and reporting with adequate human resource allocation.

## Competing interests

The authors declare there were no competing interests.

## Authors’ contributions

KW conceived the research aim, and undertook the data collection, statistical analysis, and drafting of manuscript. MRG was involved in the critical analysis and drafting of this manuscript. Both authors read and approved the final version.
